# Integration and regulation of glomerular inhibition in the cerebellar granular layer circuit

**DOI:** 10.3389/fncel.2014.00055

**Published:** 2014-02-25

**Authors:** Lisa Mapelli, Sergio Solinas, Egidio D'Angelo

**Affiliations:** ^1^Department of Brain and Behavioral Sciences, University of PaviaPavia, Italy; ^2^Brain Connectivity Center, C. Mondino National Neurological InstitutePavia, Italy

**Keywords:** synaptic inhibition, GABA receptors, granule cells, Golgi cells, cerebellum

## Abstract

Inhibitory synapses can be organized in different ways and be regulated by a multitude of mechanisms. One of the best known examples is provided by the inhibitory synapses formed by Golgi cells onto granule cells in the cerebellar glomeruli. These synapses are GABAergic and inhibit granule cells through two main mechanisms, phasic and tonic. The former is based on vesicular neurotransmitter release, the latter on the establishment of tonic γ-aminobutyric acid (GABA) levels determined by spillover and regulation of GABA uptake. The mechanisms of post-synaptic integration have been clarified to a considerable extent and have been shown to differentially involve α1 and α6 subunit-containing GABA-A receptors. Here, after reviewing the basic mechanisms of GABAergic transmission in the cerebellar glomeruli, we examine how inhibition controls signal transfer at the mossy fiber-granule cell relay. First of all, we consider how vesicular release impacts on signal timing and how tonic GABA levels control neurotransmission gain. Then, we analyze the integration of these inhibitory mechanisms within the granular layer network. Interestingly, it turns out that glomerular inhibition is just one element in a large integrated signaling system controlled at various levels by metabotropic receptors. GABA-B receptor activation by ambient GABA regulates glutamate release from mossy fibers through a pre-synaptic cross-talk mechanisms, GABA release through pre-synaptic auto-receptors, and granule cell input resistance through post-synaptic receptor activation and inhibition of a K inward-rectifier current. Metabotropic glutamate receptors (mGluRs) control GABA release from Golgi cell terminals and Golgi cell input resistance and autorhythmic firing. This complex set of mechanisms implements both homeostatic and winner-take-all processes, providing the basis for fine-tuning inhibitory neurotransmission and for optimizing signal transfer through the cerebellar cortex.

## Introduction

**Figure 1:**
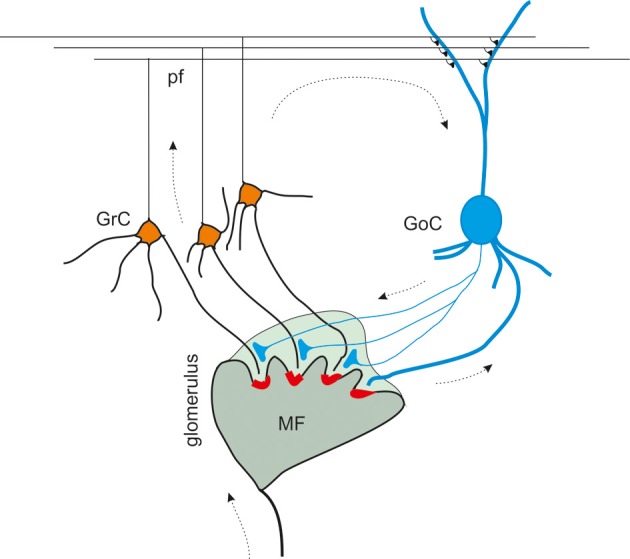
**The inhibitory circuit of cerebellar granular layer.** In the granular layer of the cerebellar cortex, granule cells receive excitatory inputs from the mossy fibers and are inhibited by Golgi cells. The synaptic contacts among granule cell (GrC) dendrites, mossy fiber (MF) terminals, and Golgi cell (GoC) axon and dendrites are enwrapped into a glial sheet, originating a peculiar anatomical structure known as cerebellar glomerulus. Each glomerulus is characterized by one mossy fiber rosette and several granule cell and Golgi cell dendrites, as well as Golgi cell axons. Each granule cell dendrite contacts different glomeruli, receiving inputs from different mossy fibers. GrC axon ascends in the molecular layer, where it originates the parallel fibers (pf), that form excitatory synapses on GoC dendrites (Palkovits et al., 1971; Palay and Chan-Palay, 1974; Hámori and Somogyi, 1983). Mossy fiber terminals contact granule cell dendrites as well as Golgi cells, that therefore inhibit granule cells in a feedforward loop. Parallel fibers originating from granule cell axons, activates Golgi cells, giving rise to a feedback inhibition on granule cells.

The fundamental anatomical and functional organization of the cerebellar cortex was defined since the ‘60s, thanks to improvements in anatomical and physiological techniques applied to the brain tissue (Eccles, 1967; Palay and Chan-Palay, 1974). Golgi cells were shown to receive excitatory inputs from mossy fibers and parallel fibers and to inhibit granule cells. Granule cell excitation and inhibition were shown to occur inside the glomeruli, where each granule cell is contacted by mossy fiber terminals and in turn receives synapses from Golgi cells (Figure 1) (Hámori and Somogyi, 1983; Jakab and Hámori, 1988). In 1964, Eccles, Llinas and Sasaki were the first to discover the GABAergic inhibitory nature of the Golgi cell-granule cell connection (Eccles et al., 1964), and the precise timing of excitation and inhibition in the circuit was reported (Eccles, 1967). A complete review on the history of Golgi cell morphological and functional studies has been presented recently (Galliano et al., 2010).

At the present state, the granular layer inhibitory circuit appears to be organized as follows. The mossy fibers, which have been shown to convey protracted discharges or short high-frequency bursts (Kase et al., 1980; Chadderton et al., 2004; Rancz et al., 2007; Arenz et al., 2008), activate both granule cells and Golgi cells inside the glomerulus. Golgi cells participate both in a feedforward loop and a feedback loop. In the feedforward loop, mossy fibers activate granule cells and Golgi cells (Kanichay and Silver, 2008; Cesana et al., 2013), which in turn inhibit the granule cells. In the feedback loop, granule cells ascending axon and parallel fibers activate Golgi cells, which in turn inhibit the granule cells (Palay and Chan-Palay, 1974; D'Angelo, 2008; Kanichay and Silver, 2008; Cesana et al., 2013; D'Angelo et al., 2013).

Most of the interest has been recently devoted to understand two components of inhibition, called *phasic* and *tonic*. While phasic inhibition was observed quite early to generate typical inhibitory post-synaptic currents (IPSCs) (Puia et al., 1994; Kaneda et al., 1995; Wall and Usowicz, 1997; Rossi and Hamann, 1998) and potentials (IPSPs) (Armano et al., 2000), growing interest has recently been raised by a tonic form of inhibition, mediated by high-affinity extrasynaptic GABA-A receptors activated by low ambient GABA concentrations in the extracellular space of the glomerulus (Brickley et al., 1996; Wall and Usowicz, 1997; Hamann et al., 2002; Rossi et al., 2003; Farrant and Nusser, 2005; Orser, 2006; Vizi and Mike, 2006; Glykys and Mody, 2007; Mohr et al., 2013). While the dynamic nature of cerebellar computations and the exceptional temporal sensitivity of cerebellar circuits require high-precision phasic inhibitory mechanisms (D'Angelo and De Zeeuw, 2009), tonic inhibition can exert additional roles. Among these, it can set the level of neuronal excitability (Brickley et al., 1996) and regulate the gain of excitatory neurotransmission at the mossy fiber-granule cell synapse (Mitchell and Silver, 2000a,b, 2003). Recent reviews focused on the origin and consequences of tonic inhibition (Rossi et al., 2003; Farrant and Nusser, 2005; Orser, 2006; Vizi and Mike, 2006; Glykys and Mody, 2007). In this review, we will consider the interplay of tonic and phasic inhibitory mechanisms and how these same are integrated with metabotropic control systems operating over all the elements of the cerebellar granular layer circuit.

## Phasic inhibition

**Figure 2:**
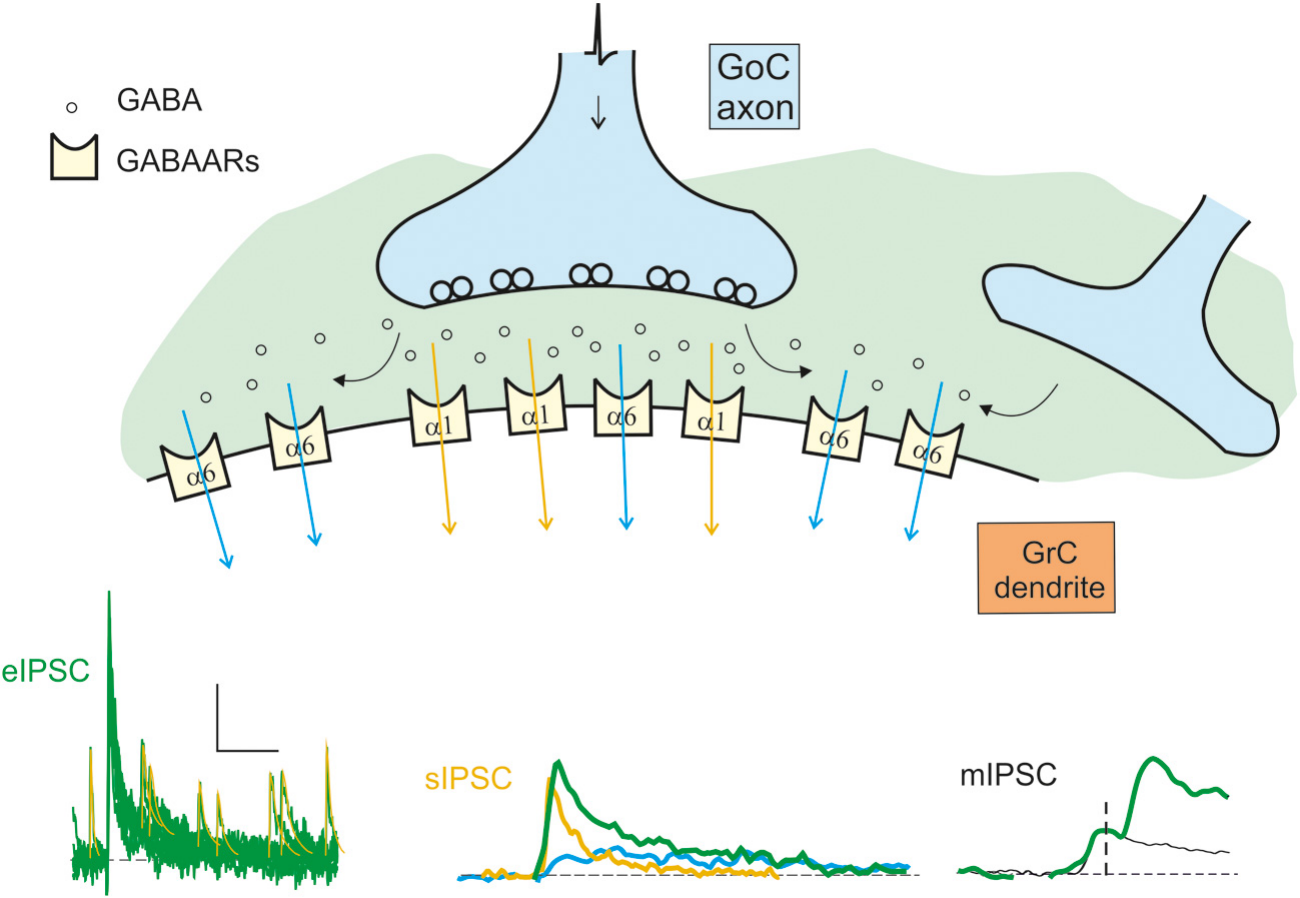
**Schematic representation of the origin of the inhibitory currents.** The schematic drawing represents the GoC to GrC synapse, illustrating the major components of inhibitory transmission. Depending on whether the α1 (synaptic) or the α6 (extrasynaptic) receptors are activated, the current give raise to the direct (fast) or indirect (slow) IPSCs. The distribution of the α1 and α6 subunit-containing GABA-A receptors (GABAARs) is indicated. Depending on the combinations of direct and indirect components, miniature (mIPSCs), spontaneous (sIPSCs), and evoked (eIPSCS) IPSCs are generated: mIPSCs are determined by random release of single quantum of neurotransmitter, sIPSCs are generated by spontaneous multiquantal release from a single synapse, eIPSCs are multiquantal multisynaptic responses. The fast direct IPSC is mediated by α1 containing receptors (yellow trace and arrows). The slow indirect IPSC is mediated by α6 containing receptors (blue trace and arrows). The resultant eIPSC (green trace) is the sum of the slow and fast currents. The scale bar is 10 pA and 100 ms for eIPSC, 10 ms for sIPSC, and 1.5 ms for mIPSC. Traces modified from Mapelli et al., 2009.

Phasic inhibition is generated by the action potential-dependent and calcium-dependent release of GABA from Golgi cell pre-synaptic terminals. This GABA generates IPSCs with complex properties depending on the coexistence of two kinetically and molecularly distinct components (Figure 2). GABA can act directly on the receptors facing the post-synaptic site (fast direct component) or indirectly on extrasynaptic receptors through spillover from the neighboring sites (slow indirect component). The direct component raises in about 1 ms and decays with a time constant around 30 ms (Rossi and Hamann, 1998; Rossi et al., 2003; Mapelli et al., 2009). The indirect component raises in several ms and decays with a time constant around 800 ms (Rossi and Hamann, 1998; Hamann et al., 2002). Direct release from the pre-synaptic terminal determines the activation of α1-containing GABA-A receptors and, in part, of α6-containing GABA-A receptors. While α1 receptors are located in the PSD, α6 receptors are located preferentially at extrasynaptic sites (but in part also in the PSD, Nusser et al., 1995). Fast kinetics of the direct component are explained by rapid gating of α1-containing GABA-A receptors. Conversely, slow kinetics of the indirect component are explained by slow gating of α6-containing GABA-A receptors. The slow receptor activation caused by diffusion of GABA to extrasynaptic receptors is in matching with their slow gating kinetics.

The minimal GABAergic responses measurable from granule cells are the miniature synaptic currents, or *mIPSCs*, which correspond to release of single neurotransmitter quanta and occur rarely, probably reflecting the small number of inhibitory synapses per granule cell. The Golgi cells are spontaneously active (Dieudonne, 1998; Forti et al., 2006; Solinas et al., 2007a,b) and generate spontaneous multiquantal inhibitory post-synaptic currents (*sIPSCs*) in granule cells (Rossi and Hamann, 1998; Mapelli et al., 2009; Brandalise et al., 2012; Hull and Regehr, 2012) with an average frequency around 4 Hz / Golgi cell (Mapelli et al., 2009). Since sIPSCs reflect activation at single Golgi connections, sIPSCs are examples of direct release without spillover from neighboring contacts. Indeed, the decay of these currents is faster than that of IPSCs evoked by electrical stimulation of the Golgi cell axon, the *eIPSCs* (Rossi and Hamann, 1998; Mapelli et al., 2009), which are composed by both a *direct component* due to release of GABA from the pre-synaptic site facing the post-synaptic density and an *indirect component* due to spillover of neurotransmitter from neighboring pre-synaptic sites.^1^

### The quantal nature of IPSCs

The Golgi to granule cell synapse was shown to conform to a general model of multiquantal neurotransmission^2^, in which sIPSC and eIPSC amplitude fluctuations are mostly generated by the variable number of quantal events occurring at multiple releasing sites (Edwards et al., 1990; Cherubini and Conti, 2001). An initial estimate of *quantum size* and *quantum variance* could be obtained from *mIPSCs* (Mapelli et al., 2009). Three independent estimates (classic binomial statistics, multiple probability fluctuation analysis and failure rate) of the quantal parameters *p*, *n*, and *q* yielded similar results. The *mIPSCs* are most likely monoquantal, with a conductance of 214 pS, a single channel conductance of 30 pS and a GABA channel open probability of 0.6 (Farrant and Nusser, 2005). By combining EM immunolocalization of GABA receptors with peak fluctuation analysis of single-site IPSCs, it was estimated that around 10 GABA-A α1 subunit-containing channels were present in the post-synaptic density and about 80% of them were open at peak, consistent with estimates of channel open probability in a saturated PSD (Nusser et al., 1995, 1998). With these parameters, a quantum is associated with an intrasite variance (cvI-ss) of 0.16. Assuming independent release of multiple quanta, MPFA yields an average release probability *p* = 0.32 and an average number of release sites *n* = 4.7; putative monosynaptic evoked responses are bi-quantal, with *m* = 2 (Mapelli et al., 2009). The small size of the inhibitory synaptic currents should not be surprising. The single quantum conductance (214 pS) is relatively large compared to the resting whole-cell conductance (around 1000 pS; D'Angelo et al., 1995; Rancz et al., 2007), so that even a single quantum can determine remarkable inhibitory effects contrasting depolarization caused by excitatory synapses (Mapelli et al., 2009).

A large variability between individual GABAergic connections has been reported concerning synaptic potency, failure rate, current time course, and quantal parameters (Crowley et al., 2009; Mapelli et al., 2009). The differences in the number of synaptic Golgi cell-granule cell contacts within a glomerulus (Jakab and Hámori, 1988) and the possibility that Golgi cells contact glomeruli on multiple dendrites provide a plausible explanation for the experimental variability. Moreover, slow IPSCs generated by GABA spillover from nearby synapses (Rossi and Hamann, 1998) have been observed (Crowley et al., 2009; Mapelli et al., 2009) suggesting that not all granule cells in a glomerulus are contacted by Golgi cell axons (Jakab and Hámori, 1988). It has been proposed that the large variability among Golgi cell responses might compensate for the lack of multiple interneuron types in the granular layer (Crowley et al., 2009).

Finally, it is worth noting that quantal parameters *p*, *n* and *q* are similar in Golgi cell-granule cell connections (Mapelli et al., 2009) and in mossy fiber-granule cell connections (Sola et al., 2004), ensuring an appropriate excitatory/inhibitory balance and similar quantal noise levels in both synaptic contacts.

### The slow component of phasic inhibition

The slow IPSC component prolongs the decay of eIPSCs and increase the time-window for synaptic integration. The diffusion of neurotransmitter from neighboring releasing sites allows intersynaptic crosstalk increasing signal divergence and the integrative capabilities of the cerebellar glomerulus. It should be noted that, when both direct and indirect IPSC components are present, the inhibitory charge carried by the α6-mediated component largely exceeds that carried by the α1-mediated component (Rossi and Hamann, 1998; Mapelli et al., 2009). The spillover-mediated activation of extrasynaptic receptors has been analyzed in detail with respect to glomerular geometry. It has been estimated that, a few milliseconds after vesicular release, the GABA concentration 2–3 μm away from the release site is sufficient to activate 16–81% of the α6-containing GABA-A receptors, while producing only 0–2% activation of α1-containing receptors (Saxena and Macdonald, 1996; Rossi and Hamann, 1998). This model reflects the fact that GABA concentration at 2–3 μm from the release site proved sufficient to substantially activate high-affinity α6 subunit-containing receptors, while α1 subunit-containing receptors require much higher GABA concentrations achieved inside the synaptic cleft in front of releasing sites.

Recently, spillover has been investigated in detail in different brain areas, in particular in the hippocampus. Due to its prolonged action, it has been proposed that spillover can modulate synaptic plasticity through the activation of GABA-B receptors and contribute to setting theta oscillation (Capogna and Pearce, 2011). In the cerebellar glomerulus, these effects remain to be investigated.

## Tonic inhibition

The first reports on electrophysiological effects related to a tonic GABA-A receptor-mediated conductance came from recordings in developing neurons in hippocampus (Valeyev et al., 1993; Ben-Ari et al., 1994) and cerebellum (Kaneda et al., 1995). In these experiments, the addition of GABA-A receptor blockers reduced the holding current indicating effects independent from conventional IPSCs (Otis et al., 1991). In the cerebellar glomerulus mossy fibers terminals, Golgi cell axons and granule cell dendritic endings are enwrapped into a glial sheet limiting molecule diffusion. It is thought that this property plays a critical role for the development of an ambient GABA concentration in the order of 0.1–0.2 mM (Santhakumar et al., 2006), sufficient to activate extrasynaptic GABA-A receptors containing α6 subunits (Saxena and Macdonald, 1996). The origin of a tonic GABA level (or “ambient GABA level”) in the cerebellum is still debated and the underlying mechanisms remain in part speculative.

### Mechanisms generating the tonic current

The mechanisms of tonic GABA-A receptor activation in the cerebellar glomerulus have been long debated^3^. A first issue is that the tonic current could involve different mechanisms depending on the developmental stage. During development the tonic current was reported to reflect the occurrence of sIPSCs caused by neurotransmitter released from Golgi cell terminals (Wall and Usowicz, 1997), while in adult animals it appeared to be independent from sIPSCs. Actually, tonic GABA level in adult animals were TTX-insensitive and calcium-independent, and were proposed to involve GABA release from Golgi cells terminals or astrocytes (Rossi et al., 2003) but not GABA release through swelling-activated channels or through reversal of the GABA transporters GAT-1 and GAT-3. Recently, the contribution of sIPSCs to determine the ambient GABA level (Rossi and Hamann, 1998) has been re-evaluated leading to the conclusion that a Golgi-dependent component of the tonic current is indeed present also in mature animals (Diaz et al., 2013). A second issue is that GABA may not be the only transmitter active on GABA-A receptors. Actually, enzymatic degradation of extracellular GABA in cerebellar slices reduced the tonic current by 30% (Wall, 2002), suggesting that high affinity GABA-A receptor subunits could be activated by taurine or beta-alanine (Rossi et al., 2003). A third issue is that tonic GABA levels could be regulated by neuromodulators. Indeed, acetylcholine (Ach) proved able to promote GABA-A receptor activation through non-vesicular GABA release generating a non-negligible component of the tonic GABA conductance (Rossi et al., 2003).

Recently, support has been provided for the proposal that the principal source of ambient GABA in the cerebellar glomerulus is represented by glial cells. GABA permeates through the Bestrophin 1 (Best1) anion channel implicated in glutamate release from astrocytes (Lee et al., 2010). Not only Best1 permits the flow of GABA molecules, but in physiological conditions its rate of activity is ideal to determine the concentration range believed to characterize tonic inhibition (around 160 nM; Santhakumar et al., 2006). Nevertheless, this finding has been questioned (Diaz et al., 2011), and further studies are needed to clarify this aspect.

## GABA-A receptor subunits involved in phasic and tonic inhibition

GABA-A receptors are pentameric assemblies usually made up from at least three different proteins from 19 different subunits (Olsen and Sieghart, 2008). These include α1−6, β1−3, γ1−3, δ, ε, θ, π (association of ρ1−3 subunits with GABA-A receptors is unclear, while it usually occurs with GABA-C receptors) (Whiting, 2003; Olsen and Sieghart, 2008, 2009). The regional and developmental receptor expression pattern is finely regulated by subunit gene expression, alternative splicing, post-translational modifications, and by the existence of various subunit assembly rules and anchoring/trafficking mechanisms (Luscher et al., 2011; Vithlani et al., 2011). It has been shown that, in mature cerebellar granule cells, the extrasynaptic α6βδ subunit-containing GABA-A receptors are critical to mediate tonic inhibition, while synaptic α1βγ2 subunit-containing receptors are most relevant to mediate direct synaptic transmission (Farrant and Nusser, 2005).

The slow eIPSC component is mediated by α6 subunit-containing GABA-A receptors, but it is still to be clarified if these receptors are the same extrasynaptic GABA-A receptors that mediate the tonic current (Brickley and Mody, 2012).

The α1 and α6 are the only α-subunits expressed in the cerebellum. The α6β 2, 3δ combination is commonly found in granule cells, which also contain the highest density of δ subunit in the cerebellum. The α6β2, 3δ, with a GABA EC50 (concentration of half-maximal activation) around 0.2 μM shows amongst the highest GABA affinities (Saxena and Macdonald, 1996) and is almost exclusively found at extrasynaptic locations (Nusser et al., 1999; Sassoè-Pognetto et al., 2000). Although it cannot be excluded that tonic conductance and/or slow IPSCs are mediated by the GABA-A receptors containing α6, β 2, and γ2, which have an EC50 of 2 μ M (Saxena and Macdonald, 1996; Cavelier et al., 2005), the δ subunit seems to be responsible for most of the tonic conductance in the cerebellum and to preferentially co-assemble with α6 subunit in the granule cells (Jones et al., 1997). Its role has been tested directly by δ subunit genetic deletion in knockout mice (Stell et al., 2003): in these animals, tonic inhibition in the cerebellum was largely reduced. In contrast with previous observations (Saxena and Macdonald, 1994), a recent paper suggested that the ambient GABA level could induce a partial desensitization of δ-containing extrasynaptic GABA-A receptors during the transient increase in ambient GABA due to spillover (Bright et al., 2011). The same results also suggested that tonic and spillover currents could be mediated by different GABA-A receptor populations.

## Functional implications of inhibition in the cerebellar glomerulus

GABA-A receptors incorporate a channel permeable to anions, in particular Cl^−^ ions. In granule cells, the GABA-A reversal potential measured using perforated-patch recordings is around −65 mV (Armano et al., 2000), i.e., it corresponds to the resting membrane potential of these neurons (D'Angelo et al., 1995). The IPSPs exert a strong inhibitory effect on granule cells limiting the excitatory post-synaptic potentials (EPSPs) rather than by hyperpolarizing the cell. Likewise, in response to step current injection from a constant holding potential, the spike input-output relationship of granule cells is left-shifted when the tonic current is blocked (Brickley et al., 1996; Hamann et al., 2002). Therefore, GABAergic inhibition operates mostly through a shunting mechanism.

The impact of the different components of inhibition are better understood by considering the different regimens of activity characterizing the mossy fiber-granule cell-Golgi cell circuit. Mossy fibers transmit bursts or long sequences of spikes and both granule and Golgi cells are able to generate bursts, in response to mossy fiber inputs (Edgley and Lidierth, 1987; Vos et al., 1999; Chadderton et al., 2004; Holtzman et al., 2006; Rancz et al., 2007; Solinas et al., 2007b). In response to mossy fiber bursts, the mossy fiber-granule cell-Golgi cell circuit engages several non-linear voltage-, time-, and frequency-dependent mechanisms leading to control different aspects of signal transmission and coding.

### Effects of the phasic inhibition

The organization of granular layer connections generates feedback and feedforward circuits that convey phasic inhibition to granule cells (D'Angelo, 2008; D'Angelo et al., 2013) (see Figure 1). The fast and the slow components of IPSCs may affect synaptic integration in different ways. In response to a mossy fiber burst, the fast component determines a sharp raise of inhibition capable of regulating the probability of emission of the first granule cell spikes, while the slow component dampens the continuation of the discharge (Crowley et al., 2009). The slow IPSC component by being associated with a slow but transient increase in GABA concentration in the glomerulus, may also act via spillover activating GABA-B receptors on both pre- and post-synaptic sites (Capogna and Pearce, 2011) (see below). Phasic inhibition plays a key role in determining various features of signal processing and synaptic integration and plasticity at the mossy fiber-granule cell relay.

**Figure 3:**
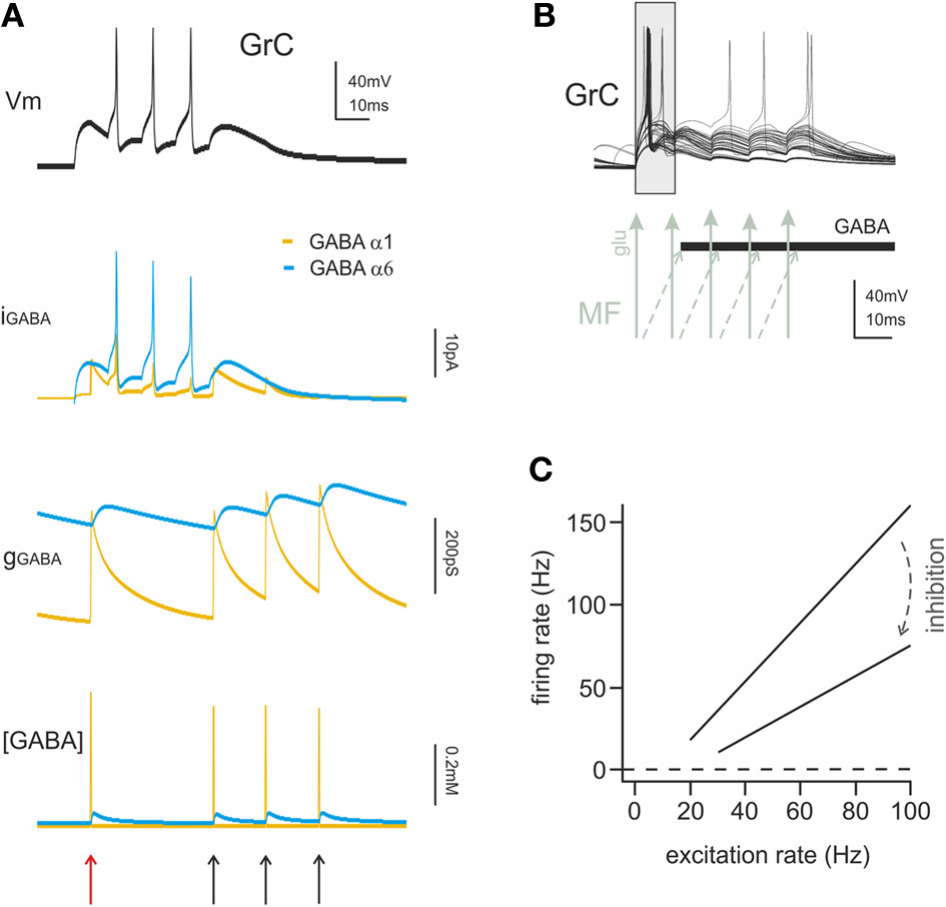
**The action mechanisms of phasic and tonic inhibition. (A)** Computational simulation of a single GrC placed in a large network model of the cerebellum cortex (15000 GrCs, 450 GoCs considering all GoCs providing input on the simulated GrCs). The GrC is located at the edge of the spot of MF terminals activated by a spike burst (5 spikes at 100 Hz), in the area of transition between the center and surround map of the excitatory/inhibitory balance. The stimulus is conveyed to the GrC by 3 of its MF synapses. The same GrC receives inhibitory input from 4 distinct GoCs, one active at the beginning of the stimulation and three activated by feedback at the end of the burst. The GrC membrane potential (*top black trace*) shows the GrC response to the stimulus. The late onset of the first spike caused by the feedforward inhibition (*red arrow*) and the generation of late spikes is prevented by the feedback inhibition (*black arrows*). The other traces provide additional information on the time course of the inhibitory input showing the total GABAergic current and conductance received by the GrC. The slow α6 component is little modulated by incoming spikes but the spontaneous activity of GoCs (8 Hz) ensures the maintenance of a basic level of inhibitory conductance. The lower trace shows the total concentration of GABA present in the synaptic cleft of the 4 inhibitory synapses. **(B)** Computational simulation of multiple granule cell spiking activity in presence of inhibition, activated through the feedforward mechanisms and generating the time-window effect. This set of GrCs was located in the center of the activated spot (where the excitatory/inhibitory balance favors excitation) and received the spike burst on all 4 of their dendrites. In this configuration the *time-window* effect dominates the GrC response favoring spikes elicited in the early phase and suppressing spikes in the late phase of the stimulus. **(C)** linear fit for granule cell I/O relationship in control and with tonic inhibition active (*arrow*), derived from dynamic clamp on acute slices. The change in the slope of the fitting indicates a change in the gain of synaptic transmission at the mf-GrC synapse (modified from Mitchell and Silver, 2003).

Phasic inhibition is critical for *time-windowing* (Figure 3A) (D'Angelo and De Zeeuw, 2009). After a mossy fiber bursts, inhibition generated by the feedforward loop reaches granule cells in 4–5 ms, thereby limiting the duration of granule cell discharge. Time-windowing allows the granule cell to fire just a few spikes. Long-term synaptic potentiation and depression (LTP and LTD) in granule cells, by regulating EPSP temporal summation and the time required to reach spike threshold in granule cells (Nieus et al., 2006), provides a very efficient mechanism to determine the spike number and frequency within the permissive time-window. It should be noted that, during prolonged mossy fiber discharges, Golgi cell inhibition on granule cells behaves differently, and generates a sustained regulation of firing frequency rather than a transmission block. It is possible that inhibition is controlled by mechanisms modifying its strength depending on the activity patterns. There are different possible candidates to operate such a regulation involving metabotropic glutamate and GABAergic receptors on mossy fiber terminals, granule cells, and Golgi cells (see below).

Phasic inhibition is critical, in conjunction with the feedback architecture of the granular layer circuit, for generating and sustaining *coherent oscillations* (Maex and De Schutter, 1998; Solinas et al., 2010). Rhythmic activity is a fundamental property of neuronal networks and is strongly dependent on phasic inhibition cyclically reverberating into the circuit. In neocortical and hippocampal circuits, inhibition plays a key role in originating theta and gamma frequency oscillations (Cobb et al., 1995; Freund, 2003; Jonas et al., 2004) thanks to mutual interconnection of interneurons by chemical and electrical synapses (Cobb et al., 1995; Galarreta and Hestrin, 2001). In the cerebellar cortex, Golgi cell autorhythmic activity (Forti et al., 2006), stellate cell-Golgi cell connections (Casado et al., 2000), Golgi cell-Golgi cell inhibitory synapses (Hull and Regehr, 2012) and Golgi cell-Golgi cell gap-junctions (Vervaeke et al., 2010) contribute to generate circuit oscillations (Maex and De Schutter, 1998; D'Angelo and De Zeeuw, 2009; Solinas et al., 2010; D'Angelo et al., 2013). A recent paper addressed the implications of inhibition in the phenomenon of resonance in the granular layer (Gandolfi et al., 2013). Granular layer resonance occurred with two peaks at 5 Hz and 7 Hz: inhibition (probably both tonic and phasic) enhanced the peak at 5 Hz but was not required for resonance to emerge. Therefore, while the block of inhibition prevented oscillations, it did not prevent (but simply modulated) resonance, indicating that the two processes have a complex and partially distinct mechanistic relationship with the inhibitory circuit.

### Effects of tonic inhibition

Tonic inhibition was shown to reduce the input resistance and membrane time constant of granule cells and to stabilize membrane potential around its resting value. An increase in tonic GABA-A current determines a reduction of the time window over which synaptic integration occurs, an increase of the firing threshold, a reduction of firing frequency, and a reduction of transmission gain (Figure 3B) (Hamann et al., 2002; Mitchell and Silver, 2003; D'Angelo and De Zeeuw, 2009). Therefore, tonic inhibition can effectively bias information processing at the mossy fiber-granule cell relay.

Theoretically, tonic inhibition is expected to affect the input/output (I/O) curve through an additive process (Hamann et al., 2002) but not the gain of signal transmission, that is a multiplicative/divisive operation (Gabbiani et al., 1994; Holt and Koch, 1997). Nevertheless, this hold true only for constant current injections, that is for stable excitatory inputs, so that gain regulation emerged when time-varying currents were injected (Mitchell and Silver, 2003). When excitation was mediated by random variations of synaptic conductance imitating excitatory neurotransmission, tonic inhibition changed the slope of the I/O relationship (Chance et al., 2002; Mitchell and Silver, 2003). Thus, tonic inhibition has a compound effect: the additive shift in I/O curve sets the basal level of granule cell excitability, while gain regulation alters the sensitivity of the neuron to changes in input frequency. In α6 KO mice the GABA-A tonic conductance is absent and its effect is partially compensated by a leakage K current, suggesting that tonic conductance regulation is indeed a relevant granule cell process (Brickley et al., 2001; Richerson, 2004).

Tonic inhibition plays a crucial role in processing rate-coded sensory information through the cerebellar cortex, both *in vitro* and *in vivo* (Mitchell and Silver, 2003; Rossi et al., 2003; Duguid et al., 2012). It has been reported that *in vivo* tonic conductance is responsible for up to 98% of the inhibitory GABAergic conductance at rest. Accordingly, tonic inhibition could effectively scale I/O granule cell transformations during sensory stimulation and reduce background firing of granule cells. In this manner, tonic inhibition would allow maintaining the proper excitability of granule cells and discriminating salient information from synaptic noise, eventually enhancing high-fidelity mossy-granule information transfer and pattern recognition in Purkinje cells (Duguid et al., 2012). Moreover, by favoring sparseness of granule cell activation, tonic inhibition would enhance the encoding of motor programs as proposed in Marr's classic motor learning theory (Marr, 1969; Albus, 1971). Finally, cerebellar granular layer modeling suggests that tonic inhibition desynchronizes granule cell oscillations generated by the Golgi cell-granule cell feedback loop during random mossy fiber input (Maex and De Schutter, 1998). Thus tonic inhibition may also impact on coherence of distributed signal processing (Singer and Gray, 1995; Semyanov et al., 2004).

### Spatial regulation of signal transmission through the granular layer

Synaptic inhibition has been recognized to play a critical role for shaping the spatial organization of granular layer activity (D'Angelo and De Zeeuw, 2009; D'Angelo et al., 2013), presumably engaging both the phasic and tonic components. The three main effects include the center-surround organization of signal transmission (Mapelli et al., 2010b), the combinatorial rearrangement of granular layer activity (Mapelli et al., 2010a), and the topographic induction of long-term synaptic plasticity at the mossy fiber-granule cell relay (Mapelli and D'Angelo, 2007).

Since inhibition has a broader distribution than excitation, when a mossy fiber bundle is discharging it generates a core of activity surrounded by an inhibition area (Mexican-hut geometry). Signals traversing the core are transmitted more fastly, with higher gain and over a broader frequency-band than in the surround. Moreover, the center tends to make LTP and the surround LTD. The superposition of multiple Mexican-huts can generate various logical operations including AND and XOR. Thus, inhibition can sculpt granular layer activity patterns over a 4D spatio-temporal domain.

## Regulation of inhibition in the cerebellar glomerulus

Since inhibition can shape the activity of neuronal networks defining their functional state and modifying signal transmission and coding, it is of extreme interest to understand how inhibition is regulated in turn. General regulation mechanisms include the turn-over and membrane expression of the different GABA receptor subunits. A major role has been proposed for gephyrin, which controls GABA receptor anchoring to the plasma membrane (Vithlani et al., 2011). Several kinases (most commonly PKC and PKA) affect extrasynaptic GABA-A receptors distribution, membrane expression and GABA sensitivity. Recent works have shown the role of protein kinases in modulating GABA-A receptor δ-subunit expression (Payne et al., 2008; Uusi-Oukari et al., 2010) and β1-containing GABA-A receptors inhibition (Connelly et al., 2013a,b) in cerebellar granule cells. Moreover, several drugs act in a receptor subunit-dependent manner. For example, furosemide is rather specific for α6-containing receptors, diazepam and zolpidem for α1-containing receptors (Rossi and Hamann, 1998; Hamann et al., 2002; Eyre et al., 2012), allowing differential pharmacological modulation of tonic and phasic inhibition (for review see Farrant and Nusser, 2005). Beside these general mechanisms, phasic and tonic inhibition in the cerebellar glomerulus are specifically regulated by modulatory and plastic processes taking place in the local microcircuit.

### Regulation of GABAergic inhibition in the cerebellar glomerulus

**Figure 4:**
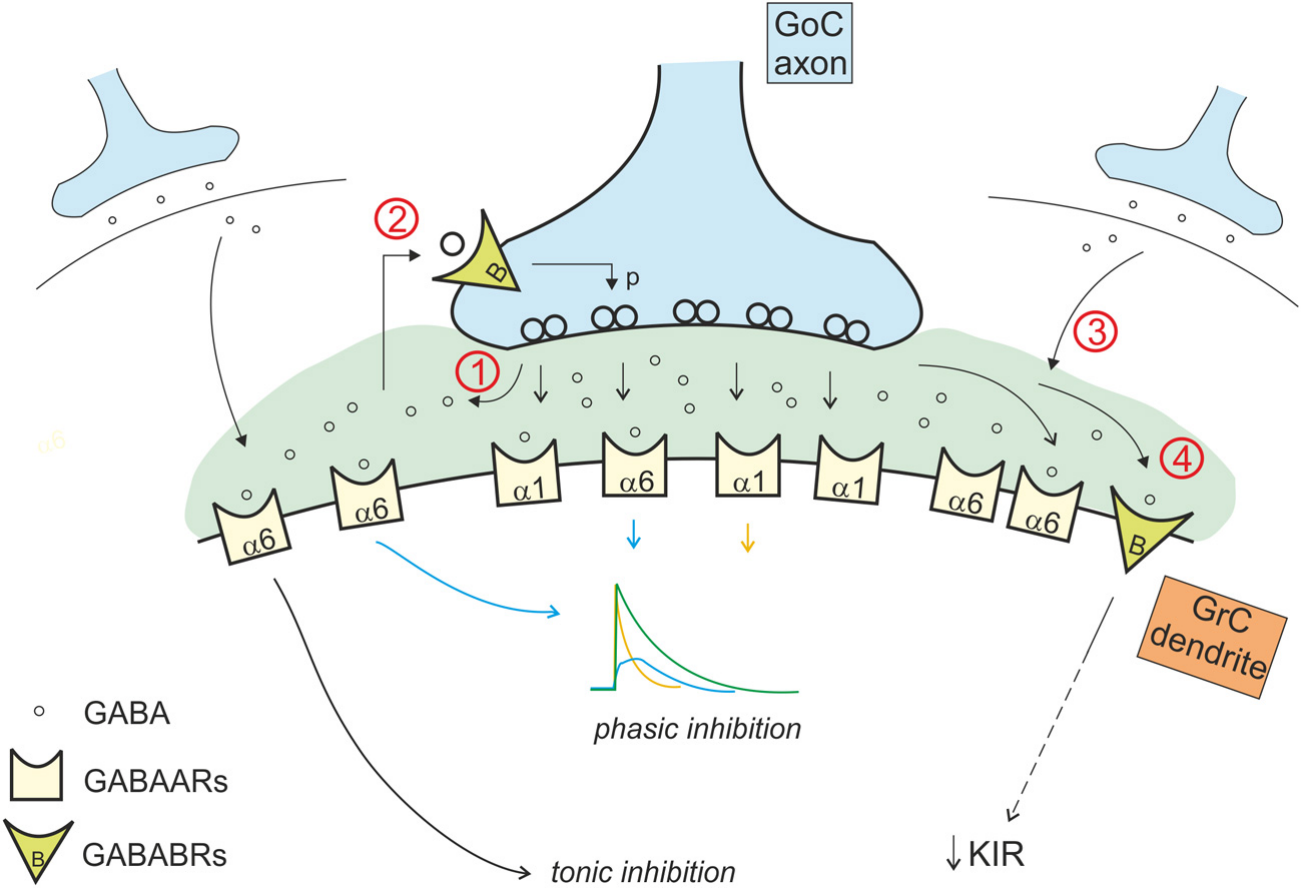
**Interaction of the phasic and tonic inhibitory mechanisms in the glomerulus.** Phasic and tonic inhibition are not independent but share a number of mechanisms and influence one each other in several ways. (1) GABA released by Golgi cell terminals during phasic inhibition contributes in increasing the level of ambient GABA, that activates extrasynaptic α6 containing GABA-A receptors, contributing to the tonic conductance (Diaz et al., 2013). (2) The tonic GABA level in the glomerulus is sufficient to activate pre-synaptic high affinity GABA-B receptors (GABABRs), that modulate release probability affecting phasic transmission (Mapelli et al., 2009). (3) GABA spillover from neighboring synapses increases the level of ambient GABA (giving a phasic contribution to tonic inhibition). (4) tonic and phasic sources of GABA (ambient and spillover) determine post-synaptic GABA-B receptors activation, modulating the K inward rectifier current and therefore granule cell excitability (Rossi et al., 2006).

In addition to activate GABA-A receptors, GABA also activates metabotropic GABA-B receptors. The GABA concentration levels attained in the cerebellar glomeruls in different phases of activity are optimal for differential GABA-B receptor activation. Pre-synaptic GABA-B receptors, which have a relatively low EC_50_ (Galvez et al., 2000; Blackburn, 2010), are present on Golgi cells synaptic terminals (Kulik et al., 2002; Mapelli et al., 2009) and are activated by ambient GABA levels (Galvez et al., 2000; Mapelli et al., 2009), around 0.01–1 μ M (Farrant and Nusser, 2005). Pre-synaptic GABA-B receptors are also present on the mossy fiber terminals (Mitchell and Silver, 2000a). Post-synaptic GABA-B receptors, which have a relatively high EC_50_ (Blackburn, 2010), are present in granule cells and are not activated by ambient GABA but rather by the high GABA levels reached during intense synaptic transmission (Galvez et al., 2000; Rossi et al., 2006; Brandalise et al., 2012). Multiple GABA-B receptor-mediated mechanisms regulating GABAergic inhibition have indeed been identified in the cerebellar glomerulus (Figure 4).

Phasic inhibition in the cerebellar glomerulus proved to be regulated by mechanisms adjusting its effectiveness with respect to past and ongoing synaptic activity. This regulation is homeostatic in nature, as it tends to reduce the impact of inhibition following intense inhibitory activity (for example a 1 s-100 Hz impulse train). In these conditions, the massive release of GABA activates post-synaptic GABA-B receptors determining downstream effects through G-protein-dependent pathways on granule cell membrane channels. A first mechanism is the long-lasting reduction of synaptic inhibition observed following intense Golgi cell-granule cell synaptic transmission, which reflects downregulation of GABA-A receptor-dependent activity (Brandalise et al., 2012). The same mechanism determined both a reduction of IPSCs and of the tonic current. A second mechanism is the long-lasting increase in granule cell excitability, which reflects reduction of the granule cell inward rectifier K^+^ current (Rossi et al., 2006). Thus, by reducing both phasic and tonic inhibition and intrinsic excitability, the excitability of granule cells is restored. This homeostatic process has important computational implication. Indeed, the number of active granule cells during signal processing has to be neither too low nor too high in order to allow optimal computation in the granular layer (Marr, 1969). Thus, the homeostatic control restoring granule cell excitability represents the functional counterpart of sparseness (Schweighofer et al., 2001).

Phasic inhibition is selectively regulated by another mechanism exploiting the effect of GABA on pre-synaptic GABA-B receptors and controlling GABA release from Golgi cell terminals. Ambient GABA generates not only the tonic current in granule cells but also a basal activation of pre-synaptic GABA-B receptors on Golgi cell synaptic terminals (Mapelli et al., 2009). Golgi cell pre-synaptic GABA-B autoreceptors determine a basal downregulation of the probability of GABA release. The mechanism shows maximal relevance at low Golgi cell activation frequencies, while it becomes irrelevant with frequencies higher than 10 Hz (Mapelli et al., 2009). This mechanism influences the first granule cell IPSC in a train, leaving unaffected the subsequent IPSCs and the overall charge transfer during the burst (Mapelli et al., 2009). This mechanism is therefore suitable to control well-timed spike transmission in granule cells rather than the continuation of busts discharge.

**Figure 5:**
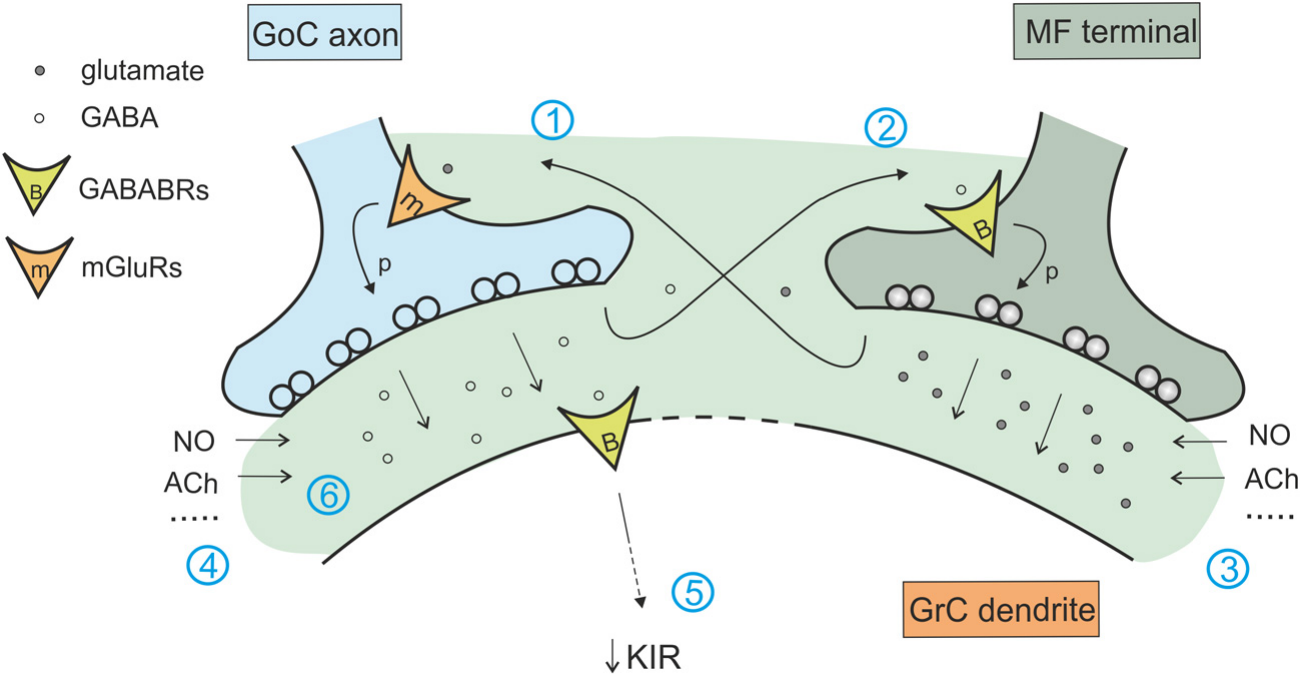
**Interaction of the excitatory and inhibitory mechanisms in the glomerulus.** The cerebellar glomerulus allows the cross-talk of excitatory mossy fiber-granule cell connections and inhibitory Golgi cell-granule cells contacts. (1) glutamate spillover to Golgi cell pre-synaptic mGluRs determines a decrease in GABA release (Mitchell and Silver, 2000b). (2) GABA spillover activates pre-synaptic GABA-B receptors on mossy fiber terminals, causing a decrease in glutamate release (Mitchell and Silver, 2000a). (3) Various modulators of excitatory transmission affect inhibition through mechanism 1, regulating the amount of glutamate released (Maffei et al., 2003; Sola et al., 2004; Prestori et al., 2013). (4) In the same way, modulators of inhibitory transmission affect glutamate release through mechanism 2 (Rossi et al., 2003; Wall, 2003; Brandalise et al., 2012). (5) Protracted inhibition activates post-synaptic GABA-B receptors and determines a decrease in K inward rectifier current, modulating granule cell excitability and its responsiveness to mossy fiber inputs (Rossi et al., 2006). (6) Modulators of tonic and phasic inhibition contributes in regulating the amount of GABA in the synaptic cleft and spilling over to mossy fiber terminals, acting on excitatory transmission through mechanisms 2 and 5.

The transient increase in GABA concentration due to synaptic activity determines also activation of pre-synaptic GABA-B receptors on mossy fiber terminals, decreasing glutamate release at mossy fiber-granule cell relay (Mitchell and Silver, 2000b). Similarly, spillover of glutamate released from mossy fibers activates pre-synaptic mGluR2,3 receptors on Golgi cells (Mitchell and Silver, 2000a). In this case, the effect is to decrease the release probability at the inhibitory connection between Golgi and granule cell (Mitchell and Silver, 2000a), providing a net excitatory action. The intensity of these effects depends on neuron firing frequency: high mossy fiber frequencies provide more glutamate enhancing the blockade of GABA release, while high Golgi cell frequency provides more GABA release enhancing blockade of glutamate release (Mitchell and Silver, 2000b). Thus this regulatory subsystem seems to be conceived to prize the winner in the competition between the different elements of the cerebellar glomerulus sharpening the transition between granule cell excitation and inhibition (Figure 5).

Yet another metabotropic control mechanism operates in the Golgi cells. It has been shown that glutamate released by granule cells at the parallel fiber-Golgi cells synapse can activate mGluR2 on Golgi cells determining hyperpolarization induced by G protein-coupled inwardly rectifying K^+^ channels (GIRKs). This leads to a long-lasting reduction of Golgi cell firing dependent on granule cell activity (Watanabe and Nakanishi, 2003). This is another homeostatic process tending to balance activity in the feedback inhibitory loop (Figure 5).

It should be noted that the mechanisms involved in regulating the ambient GABA level are themselves subject to modulation. Although GABA transporters may not provide the main source of tonic inhibition (Semyanov et al., 2003), this latter proved sensitive to GABA uptake, as suggested by the high level of tonic currents in GAT-1 deficient mice (Jensen et al., 2003). The tonic current can also be modulated by synaptic activity through activation of post-synaptic GABA-B receptors (Brandalise et al., 2012). Such an effect may reflect the inhibitory action of protein kinase A (PKA) on β 1-containing GABA-A receptors (Connelly et al., 2013b). Another possible mechanism involves the activation of Best1, that is volume-sensitive, so that swelling can trigger GABA release from the glia (Kozlov et al., 2006; Lee et al., 2010).

Several additional mechanisms can regulate GABA release and therefore the ambient GABA level and the tonic current. The tonic current in the cerebellum is modified by ACh application. ACh-evoked currents predominantly derive from vesicular release of GABA, but approximately 15–26% is generated by non-vesicular release (Rossi et al., 2003). The cholinergic innervation in the rat cerebellum is predominantly provided by mossy fibers from the vestibular nuclei to the uvula and nodulus of the vermis and by more diffusely terminating fibers from the pedunculopontine tegmental and lateral paragigantocellular nuclei (Jaarsma et al., 1997). Another possible source of modulation for the inhibitory tonic current in the granular layer is represented by nitric oxyde (NO). It has been reported that the reduction of NO levels in cerebellar slices enhances both the tonic and phasic component of inhibition; the mechanism seems to be pre-synaptic and may act by modulating GABA release probability (Wall, 2003). Finally, extrasynaptic GABA-A receptors that mediate the tonic current, are sensitive to neurosteroids, that are normally present in the intracellular space in the nanomolar concentration range, which is enough to activate δ-containing GABA-A receptors (Zheleznova et al., 2009). This property suggest a connection between these receptors and stress-, ovarian cycle- and pregnancy- related mood disorders (Brickley and Mody, 2012).

## Integrated regulation of inhibitory circuit functions

**Figure 6:**
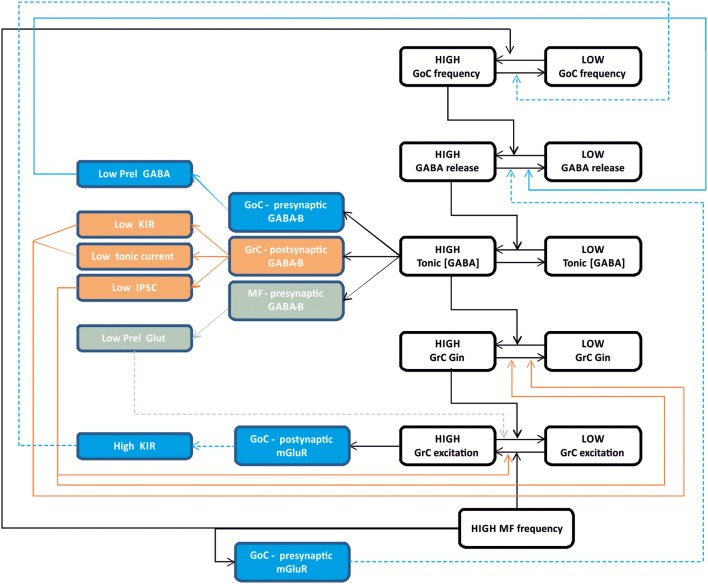
**Integrated control of inhibition in the granular layer circuit.** The flow chart represent the glomerular interaction of excitatory and inhibitory mechanisms, converging onto granule cells. The colored boxes on the left represent the modulating (dashed lines) and homeostatic (solid lines) effects involving Golgi cells (blue), granule cells (orange) and mossy fibers (gray boxes). Modulatory and homeostatic mechanisms are initiated following high inhibitory and/or excitatory activity. In particular, tonic GABA levels control GABA-B receptors functions tending to counterbalance high inhibitory activity.

The process of synaptic inhibition in the cerebellar glomerulus integrates different aspects of ionotropic and metabotropic control involving various types of receptors and cellular elements, from pre-synaptic terminals to neurons and glial cells. The ionotropic system regulates granule cell input conductance, both tonically and phasically, effectively controlling the granule cell output. The metabotropic system regulates the balance of excitation and inhibition in the mossy fiber-granule cell-Golgi cell loop (Figure 6).

The ionotropic responses have been conveniently divided into phasic and tonic, but these appear to be deeply integrated and to subserve a common final goal, that of regulating signal transmission along the mossy fiber-granule cell pathway. Both tonic and phasic inhibition control granule cell input conductance although on different time scales. Moreover, phasic and tonic inhibition regulate one each other. The amount of synaptic GABA release contributes to determine the ambient GABA level and tonic inhibition. The ambient GABA level in the glomerulus, once established, reduces GABA release probability. Moreover, the ionotropic system can be regulated by a number of other factors controlling GABA release and reuptake.

The metabotropic system is much less known but available evidences suggest that it plays a complex regulatory role running along two different pathways controlled by the GABA and glutamate systems. The GABA system provides a major homeostatic drive coming from the ambient GABA level, whose increase reduces granule cell excitability but at the same time activates a number of mechanisms to restore it. In the glomerulus, the action passes through GABA-B receptors, which reduce (i) GABA release from pre-synaptic Golgi cell terminals, (ii) granule cell GABA-A receptor-mediated IPSCs, (iii) granule cell inward rectifier, (iv) glutamate release from mossy fiber terminals. Thus, it seems that the glomerulus employs all available mechanisms to counterbalance an excess of inhibition, which would severely damage information transfer through the mossy fiber-granule cell pathway. The glutamate system appears to operate on a different principle. In the feedback loop, the increase of glutamate released by active granule cells can activate Metabotropic glutamate receptors (mGluRs) at the parallel fiber-Golgi cell synapses, enhancing the inward rectifier current and hyperpolarizing the Golgi cell. In the glomerulus, a glutamate increase reduces GABA release from mossy fiber terminals. In both cases, Golgi cell inhibition is reduced implementing a “winner-take-all” mechanism. Thus, the final effect of inhibition on the granular layer circuit will depend on a variety of local and external factors. This aspect deserves future experimental investigation.

The inhibitory circuit could be regulated also by other intrinsic and extrinsic factors, including general neuromodulators. For example, serotonin activates Lugaro cells (Dieudonné and Dumoulin, 2000), which inhibit Golgi cells and could therefore reduce inhibition of the granule cells. NO, which is produced during high-frequency mossy fiber activity (Maffei et al., 2003), enhances GABA release and the tonic GABA level (Wall, 2003). Finally, it would be of extreme interest to understand if and how the inhibitory system of the granular layer expresses forms of long-term synaptic plasticity, as it would have a profound impact on signal coding along the mossy fiber-granule cell pathway (Garrido et al., 2013a,b). At present, there is no evidence for long-term synaptic plasticity between Golgi cells and granule cells. However, a form of LTD has been reported at the parallel fiber-Golgi cell synapse (Robberechts et al., 2010) and Golgi cells may undergo persistent changes in intrinsic excitability controlling their basal firing frequency (Hull et al., 2013). Further experiments are needed to clarify these issues.

## Conclusions

Current evidence suggests that glomerular inhibition is designed to fine-tune the excitability of granule cells in order to optimize information transfer through the mossy fiber-granule cell relay. This effect is obtained through a wide range of actions including (i) regulation of timing, number and frequency of granule cell spikes, (ii) regulation of granular layer oscillations and resonance, (iii) regulation of center-surround responses and logical operations in the granular layer, (iv) regulation of long-term synaptic plasticity at the mossy fiber-granule cell relay. These effects should be eventually integrated and converge toward the optimization of granular layer functions. The most attractive perspective is now to understand the intricate balance of mechanisms that concur to generate efficient transmission along the mossy fiber pathway, to ensure an efficient inhibition determining sparseness, and to prevent that excessive inhibition blocks transmission degrading information transfer. This investigation may be aided by mathematical models resolving the intricate network of interactions regulating glomerular and inhibitory circuit functions.

### Conflict of interest statement

The authors declare that the research was conducted in the absence of any commercial or financial relationships that could be construed as a potential conflict of interest.

## Footnotes

^1^In the granular layer, the isolation of a putative single excitatory connection between mossy fibers and granule cells is achieved considering the minimal stimulation intensity that elicits a response. In the inhibitory Golgi cell-granule cell relay, it is possible to use a minimal stimulation protocol and to compare the amplitude of the eIPSCs with that of sIPSCs. This has been done with success in a recent work, and allowed to accurately determine different parameters of direct transmission and to analyze its quantal nature (Mapelli et al., 2009)

^2^Quantal analysis is based on the binomial theory, applied first at the neuromuscular junction by Katz and Miledi in the 1965 (Katz and Miledi, 1965). The quantal theory states that the mean number of quanta released at each impulse (m, mean quantum content) depends on the number of releasing sites (n) and on the probability (p) that each quantum (q, quantum size) is released. In central synapses, the binomial theory is enlarged considering additional sources of variability, as intrasite (type I) and intersite (type II) quantum variability. Intrasite variability (cvI) reflects fluctuation in the number of open channels at single sites (cvI-ss) and asynchrony in quantal delay at the eIPSC peak (cvI-qd). Intersite variability (cvII) reflects differences among postsynaptic densities. The Multiple Probability Fluctuation Analysis (MPFA; Clements and Silver, 2000) includes this other sources of variability present at central synapses, and allows to obtain an estimate of the quantal parameters of synaptic transmission at a central synapse.

^3^There are many technical problems in studying tonic conductance. For instance, the sources of extracellular GABA have been studied in slices, where artificial factors can induce changes in the extracellular composition. The volume of the extracellular space can change depending on the region of the brain, the age of the animal and the level of oxygenation of the tissue, since hypoxia shrinks the extracellular space and could lead to an increase in GABA concentration. For the same reason, the rate of slice perfusion in the recording chamber can influence these parameters and affect the extracellular GABA concentration. The temperature of recordings affects the rate of action of GABA transporters and other processes that can take part in regulating GABA level (Glykys and Mody, 2007). The existence of a tonic current *in vivo* has been reported in the cerebellum (Chadderton et al., 2004), thus ruling out the possibility that this conductance is a pure artifact of the slicing method. The glial sheath of the cerebellar glomeruli prevents some of the previously cited artificial effects, since it protects the extracellular environment of the synaptic connections investigated. The cerebellum granular layer is then the ideal structure in which to investigate properties and functional consequences of the tonic current. Although cerebellar tonic current recorded in slices can be considered “physiological,” some doubts remain on the reliability of this model for questioning the sources of the extracellular GABA.
