# Corrigendum: Integration and regulation of glomerular inhibition in the cerebellar granular layer circuit

**DOI:** 10.3389/fncel.2014.00342

**Published:** 2014-10-20

**Authors:** Lisa Mapelli, Sergio Solinas, Egidio D'Angelo

**Affiliations:** ^1^Brain Connectivity Center, C. Mondino National Neurological InstitutePavia, Italy; ^2^Department of Brain and Behavioral Sciences, University of PaviaPavia, Italy

**Keywords:** synaptic inhibition, gaba receptors, granule cells, golgi cells, cerebellum

Sergio Solinas is affiliated exclusively with the Brain Connectivity Center, C. Mondino National Neurological Institute, Pavia, Italy.

## Conflict of interest statement

The authors declare that the research was conducted in the absence of any commercial or financial relationships that could be construed as a potential conflict of interest.

