# Corrigendum: Integration and regulation of glomerular inhibition in the cerebellar granular layer circuit

**DOI:** 10.3389/fncel.2016.00031

**Published:** 2016-02-16

**Authors:** Lisa Mapelli, Sergio Solinas, Egidio D'Angelo

**Affiliations:** ^1^Brain and Behavioral Sciences, Neurophysiology Unit, University of PaviaPavia, Italy; ^2^Brain Connectivity Center, C. Mondino National Neurological InstitutePavia, Italy

**Keywords:** synaptic inhibition, gaba receptors, granule cells, golgi cells, cerebellum

The corrigendum is needed in the acknowledgments section, since a funding agency was erroneously forgotten. We request therefore to add the sentence:

**.… and by grants of the Italian Ministry of Health (RF-2009-1475845) to ED**.

So that the full corrected paragraph will become:

## Acknowledgments

We thank Leda Roggeri for technical assistance. This work was supported by European Union grants [CEREBNET FP7-ITN238686, REALNET FP7-ICT270434, Human Brain Project (HBP-604102)] **and by grants of the Italian Ministry of Health (RF-2009-1475845) to ED**.

We confirm that missing information does not affect the scientific validity of the results at any extent.

## Author contributions

All authors listed, have made substantial, direct and intellectual contribution to the work, and approved it for publication.

### Conflict of interest statement

The authors declare that the research was conducted in the absence of any commercial or financial relationships that could be construed as a potential conflict of interest.

